# Domoic Acid-Induced Neurotoxicity Is Mainly Mediated by the AMPA/KA Receptor: Comparison between Immature and Mature Primary Cultures of Neurons and Glial Cells from Rat Cerebellum

**DOI:** 10.1155/2011/543512

**Published:** 2011-11-02

**Authors:** Helena T. Hogberg, Anna K. Bal-Price

**Affiliations:** ^1^Environmental Health Science, School of Public Health, The Johns Hopkins University, Baltimore, MD 21205-2103, USA; ^2^Center for Alternatives to Animal Testing, Bloomberg School of Public Health, Johns Hopkins University, 615 N Wolfe Street, W7032 Baltimore, MD 21205, USA; ^3^In-Vitro Methods Unit, Institute for Health and Consumer Protection, European Commission Joint Research Centre, 21020 Ispra (VA), Italy

## Abstract

Domoic acid (DomA) is a naturally occurring shellfish toxin that can induce brain damage in mammalians. Neonates have shown increased sensitivity to DomA-induced toxicity, and prenatal exposure has been associated with e.g. decreased brain GABA levels, and increased glutamate levels. Here, we evaluated DomA-induced toxicity in immature and mature primary cultures of neurons and glial cells from rat cerebellum by measuring the mRNA levels of selected genes. Moreover, we assessed if the induced toxicity was mediated by the activation of the AMPA/KA and/or the NMDA receptor. The expression of all studied neuronal markers was affected after DomA exposure in both immature and mature cultures. However, the mature cultures seemed to be more sensitive to the treatment, as the effects were observed at lower concentrations and at earlier time points than for the immature cultures. The DomA effects were completely prevented by the antagonist of the AMPA/KA receptor (NBQX), while the antagonist of the NMDA receptor (APV) partly blocked the DomA-induced effects. Interestingly, the DomA-induced effect was also partly prevented by the neurotransmitter GABA. DomA exposure also affected the mRNA levels of the astrocytic markers in mature cultures. These DomA-induced effects were reduced by the addition of NBQX, APV, and GABA.

## 1. Introduction

Mechanisms of domoic acid- (DomA-) induced toxicity have been extensively investigated since an incident in 1987 in Eastern Canada where several hundred people experienced serious health problems after ingesting mussels. DomA-induced toxicity has been studied mainly in adult animals, and fetal developmental effects have only been evaluated in a limited number of studies. Based on these few experiments, neonates have been shown to be more sensitive to DomA per body weight than adults [1–5]. The reduced serum clearance has been proposed as a contributing factor to their increased vulnerability as well as greater access of DomA through the undeveloped blood-brain barrier [1, 4]. DomA has also been shown to cross the placenta and can reach the brain tissue of the fetus and accumulate in the amniotic fluid [6]. Moreover, a higher quantity of DomA remains in the milk as compared to the plasma, and therefore, a new born baby can be more exposed than the mother [7]. Prenatal exposure to DomA has been associated with damage to neurons in different brain regions; however, the mechanisms of neurotoxicity is not entirely clear. Some studies suggest that DomA decreases levels of brain gamma-amino butyric acid (GABA) and increases glutamate levels [8]. Moreover, DomA exposed offspring have shown neurobehavioral changes that have persisted into adulthood [2, 5], such as an increase in response latency and in rate of habituation. On the contrary to *in vivo* studies, *in vitro* studies have shown increased DomA toxicity with increasing maturation of the CNS [9]. Therefore, the developmental neurotoxic effects of DomA need to be further studied to determine if this could be due to different toxic mechanisms, renal clearance, or increased exposure.

DomA is structurally related to kainic acid (KA), which is an analogue to the excitatory amino acid and neurotransmitter L-glutamate. Most likely, DomA activates the *α*-amino-3-hydroxy-5-methyl-4-isoxazolepropionic acid/KA receptor (AMPA/KA-R), which induces increased levels of intracellular Ca^2+^ which, in turn, causes glutamate release that subsequently activates the N-methyl-D-aspartic acid receptor (NMDA-R) [10, 11]. Activation of AMPA/KA-Rs (direct) and NMDA-Rs (indirect) can cause apoptotic and necrotic neuronal cell death [12, 13]. The mode of neuronal cell death, apoptotic or necrotic, in pure neuronal cultures seems to depend on the concentrations of DomA, as a low concentration (0.1 *μ*M) induces apoptosis mainly through AMPA/KA-Rs, and a high concentration (10 *μ*M) induces necrosis also through glutamate release and secondary activation of NMDA-R [14]. In addition, the exposure time has also been identified as an important factor that might increase the DomA-induced toxicity [9].

Furthermore, a few studies have suggested involvement of glial cells that could enhance the DomA-induced neurotoxicity [15, 16]. Some studies have reported that exposure of astrocytic cultures to DomA did not induce any cell death [14, 15], but it changed the glia function, as inhibition of the glutamate uptake was observed [15]. The mechanism behind the inhibition of glutamate uptake is not yet known but could be a secondary effect related to decreased levels of ATP, glutamate-receptor activation, intracellular acidification, or free-radical formation [15]. Moreover, there are to our knowledge hardly any studies performed in mixed glial-neuronal cultures, which are the most relevant models to *in vivo* situations. The interaction between glia and neurons is certainly important especially during development of the brain and could play an important role in DomA-induced toxicity both in *in vivo* and in *in vitro* systems.

In this study, we have used mixed neuronal-glial primary cultures of CGCs to determine the mechanisms of DomA-induced toxicity in both immature and mature cultures. As a main endpoint for toxicity evaluation, we have used gene expression, as in our previous studies it has been shown that the mRNA levels of different cell-type-specific markers (neuronal and astrocytic) at various time points of cell development and maturation could be a useful tool to detect developmental neurotoxicants [17, 18]. The purpose of this study was to determine if the selected genes identified as specific markers for glial and neuronal cells could serve as an endpoint for *in vitro* assessment of DomA-induced toxicity. Furthermore, we evaluated if developing (immature) and mature mixed neuronal-glia cultures of primary rat cerebellar granule cells (CGCs) were affected in different ways by domoic acid exposure. Indeed, the mRNA expressions of the neuronal and astrocytic markers were altered after the exposure to DomA in both immature and mature cultures. Interestingly, the mature cultures seemed to be more vulnerable than the immature ones, probably due to the higher expression of NMDA and AMPA receptors. In both cell culture models, the DomA effect was mainly mediated through the AMPA/KA-R. However, not only the antagonist for AMPA/KA-R, but also antagonist of the NMDA-R and neurotransmitter GABA reduced the DomA-induced changes.

## 2. Materials and Methods

### 2.1. Chemicals and Reagents

Reagents for cell culture were purchased from Gibco Invitrogen (Milano, Italy); DMEM, Fetal Bovine Serum, Horse Serum, L-Glutamine, Gentamicin, Versene, Hepes and from Sigma-Aldrich (Milano, Italy); Poly-L-Lysine, D (+) Glucose, Potassium chloride, domoic acid, 1, 2, 3, 4-tetrahydro-6-nitro-2, 3-dioxo-benzo[f]quinoxaline-7-sulfonamide (NBQX), ((2*R*)-amino-5-phosphonovaleric acid (APV) and GABA.

### 2.2. Primary Culture of Rat Cerebellar Granule Cells (CGCs)

The primary cultures of cerebellar granule cells (CGCs) were prepared from 7-day old Wistar rat pups as described previously [19]. The cerebella were dissociated in Versene solution (1 : 5000) and plated at 0.25 × 10^6^ cells/cm^2^ in 12- or 96-well Costar plates coated with poly-L-lysine (0.01% diluted 1 : 10 (v/v) in sterile MilliQ water). Cultures were maintained in DMEM supplemented with 5% heat inactivated horse serum, 5% heat inactivated fetal bovine serum, 13 mM glucose, 0.5 mM HEPES buffer, 2 mM L-glutamine, 25 mM KCl and 10 *μ*g/mL gentamicin. Cells were maintained at 37°C in a humidified atmosphere of 5% CO_2_. The medium of CGCs was not changed throughout the whole experimental period, as these cells have to be cultured in self-conditioned medium. 

### 2.3. Domoic Acid Treatment of CGCs

The concentrations of domoic acid were chosen based on preliminary range-finding experiments, where wide ranges of concentrations were tested using the Alamar Blue (AB) (resazurin, Sigma, Milano, Italy) cell viability assay (data not shown). In the final experiments three noncytotoxic concentrations (5, 10, and 20 *μ*M) were selected based on the AB assay results. Immature cultures were exposed to domoic acid twenty-four hours after isolations for 3 or 10 days, to cover critical developmental processes at various stages of cell maturation. Mature cultures were exposed to DomA for 3 or 10 days at 7 DIV, when the culture is considered mature [20]. To determine whether the presence of domoic acid influenced the selected gene expression, cell samples were prepared for quantitative real-time PCR analysis before exposure (1 DIV) and after 3 or 10 days of DomA exposure in both immature mature cultures. 

### 2.4. Antagonists or Agonist Treatment of CGCs

Both immature and mature control cultures (nonexposed) and cultures exposed to 20 *μ*M domoic acid were pretreated with the AMPA/KA-R antagonist (NBQX, 20 *μ*M), NMDA-R antagonist (APV, 100 *μ*M), or the neurotransmitter GABA (10 *μ*M). The cultures were pretreated with the antagonists or GABA at 1 DIV (immature cultures) or 7 DIV (mature cultures) and thereafter added every third day of treatment. To determine whether the presence of the antagonists or GABA could prevent the domoic acid induced toxicity as measured by gene expression, mRNA was extracted from treated and nontreated cultures for quantitative real-time PCR analysis after 3 or 10 days of exposure to DomA of immature and mature cultures.

### 2.5. Assessment of Cell Viability Using Alamar Blue

Cell viability was determined after 3 and 10 days of exposure to DomA using the AB (resazurin) assay [21]. The blue colored indicator dye resazurin is reduced into fluorescent resorufin by red-ox reactions in viable cells. Resazurin (10 *μ*L of 100 *μ*M stock) in Hank's buffered salt solution was added directly to the 96-well plates, without removing the medium (100 *μ*L). The plates were incubated for 2 h at 37°C, 5% CO_2_. After incubation, the fluorescence of the resazurin metabolite, resorufin was measured at 530 nm/590 nm (excitation/emission) in a multiwell fluorometric reader (Fluoroskan Ascent, Labsystem, Helsinki, Finland). 

### 2.6. RNA Purification, Reverse Transcription, and Quantitative Real-Time PCR

Cell samples for analysis of mRNA expression were lysed, and total RNA extraction was performed according to the manufacturer's protocol of RNeasy Mini Kit (Qiagen, Milan, Italy). Possible contamination with DNA was removed by digestion using an RNase-free DNase set (Qiagen). RNA concentration and protein contamination were assessed spectrophotometrically (Biophotometer; Eppendorf, Milan, Italy). Reverse transcription was performed as follows: 500 ng RNA was incubated with 2.5 mM PCR Nucleotide Mix (Promega, Milan Andorra, Italy) and 12.5 *μ*g/mL random primers (Promega) for 5 min at 65°C using a Perkin-Elmer Geneamp PCR system 9600. Subsequently, 2 units/*μ*L RNaseOut inhibitor (Invitrogen), 10 units/*μ*L M-MLV reverse transcriptase (Promega) was added with the respective M-MLV buffer (Promega), and the samples were incubated for 10 min at 25°C for annealing, 60 min at 37°C for cDNA synthesis and 15 min at 70°C for inactivation of enzymes.

An AbiPrism 7000 sequence detector system in conjunction with TaqMan Universal PCR Master Mix and TaqMan Real-Time PCR Assays-on-Demand (Applera Italia, Monza, Italy) was used for investigating the gene expression and the house keeping gene according to the manufacturer's protocol. The primers used were 18S ribosomal RNA (18S rRNA, Hs99999901_s1) (TaqMan Gene Expression Assays ID), neurofilament, light polypeptide 68 kDa (Nfl, Rn00582365_m1), neurofilament, heavy polypeptide 200 kDa (Nefh, Rn00709325_m1), ionotropic glutamate receptor N-methyl D-aspartate 1 (GRIN1, Rn00433800_m1), ionotropic glutamate receptor AMPA1 (alpha 1) (Gria1, Rn00709588_m1), gamma-amino butyric acid A receptor delta (Gabrd, Rn01517015_g1), glial fibrillary acidic protein (Gfap, Rn00566603_m1), S100 protein, beta polypeptide (S100*β*, Rn00566139_m1), and nestin (Nes, Rn00564394_m1). Relative RNA quantification was performed using the comparative C_T_ method, normalizing the data to a standard calibrator (a mixture of samples from the different time points of the cell proliferation and differentiation), and to the 18S rRNA content [22]. 

### 2.7. Statistical Analysis

The GraphPad Prism 5.0 (GraphPad software, San Diego, Calif, USA) program was used for statistical analyses. All data given are means of three independent experiments performed in duplicates ± standard error of the mean (S.E.M.). One-way ANOVA and posttest (0.05) were performed to assess differences between different time points, and two-way ANOVA and posttest (0.05) were performed to assess differences between treated and control in the quantitative real-time PCR experiments. All data were log-transformed to achieve Gaussian distribution. Statistical significance was indicated as follows ^+^ *P* < 0.05, ^++^ *P* < 0.01 and ^+++^ *P* < 0.001 (3 days versus 10 days) and *P < 0.05, **P < 0.01 and ***P < 0.001 (treated versus control).

## 3. Results

### 3.1. Domoic Acid Exposure Downregulated the mRNA Levels of the Neuronal Cytoskeleton Proteins (NF-68 and NF-200) in both Immature and Mature Cultures

**Figure 1:**
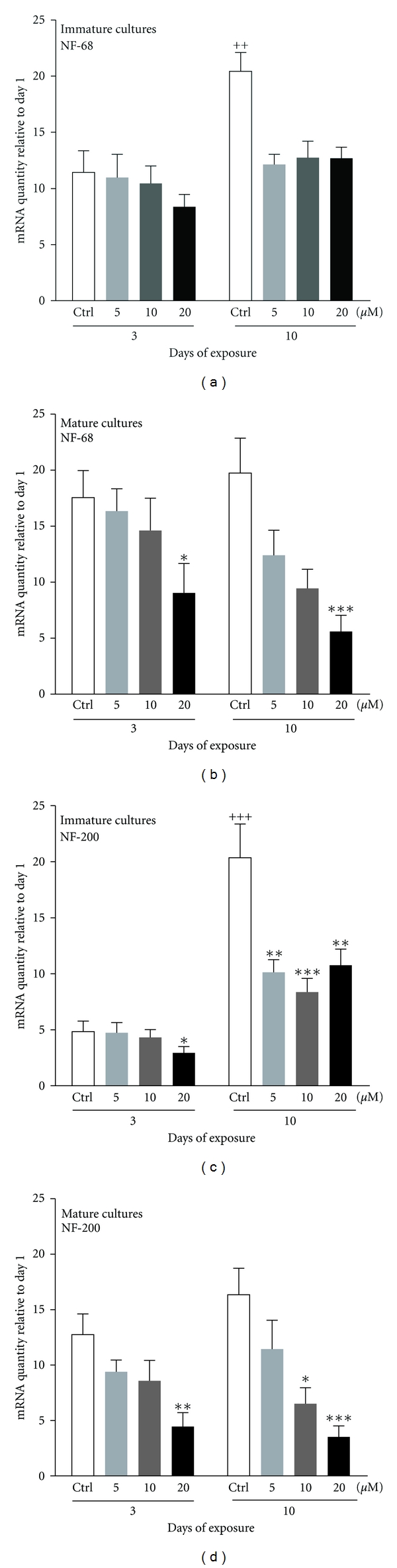
Changes in the mRNA levels of NF-68 and 200 in primary cultures of CGCs exposed to domoic acid (5 *μ*M, 10 *μ*M, and 20 *μ*M) at 1 DIV (immature cultures) or at 7 DIV (mature cultures) for 3 or 10 days. Note the significant downregulation of (a) NF-68 in immature cultures after 10 days of exposure, (b) NF-68 in mature cultures, (c) NF-200 in immature cultures, and (d) NF-200 in mature cultures. Gene expression levels were normalized to the standard calibrator, the housekeeping gene 18S rRNA and the mRNA expression at 1 DIV. Data represent mean ± S.E.M. of three independent experiments performed in duplicates. ^+^ *P* < 0.05  ^++^ *P* < 0.01  ^+++^ *P* < 0.001 comparing control cultures at 3 and 10 days. *P < 0.05  **P < 0.01  ***P < 0.001 comparing treated to control (untreated) cultures.

In order to determine if immature mixed neuronal-glial primary cultures of CGCs were more sensitive to domoic acid toxicity than mature ones, both cultures were exposed to the same range of subcytotoxic concentrations of domoic acid (5, 10 and 20 *μ*M) up to 10 days at 1 DIV (immature) or at 7 DIV (mature). These concentrations were not cytotoxic as confirmed by cell viability assay (data not shown). Two cytoskeleton proteins were selected, the neurofilament 68 (NF-68) that is the first to be expressed during the initial neurite outgrowth and neurofilament 200 (NF-200) that is expressed later and is recognized as a marker of the mature neuronal network. In control (nontreated) cultures, a significant increase in the mRNA levels for both NF-68 and NF-200 was observed with time in the immature cultures between 3 and 10 days (Figures 1(a) and 1(c)), while in the mature cultures the mRNA levels of the neurofilaments remained stable (Figures 1(b) and 1(d)).

#### 3.1.1. Effects of Domoic Acid

**Immature Cultures**

The prolonged exposure for 10 days to DomA at all studied concentrations (5, 10 and 20 *μ*M) caused a significant downregulation of both the NF-68 mRNA (by 41 ± 4%, *P* < 0.05; 38 ± 7%, *P* < 0.05; 38 ± 5%, *P* < 0.05) and the NF-200 mRNA level (by 50 ± 6%, *P* < 0.01; 59 ± 6%, *P* < 0.001; 47 ± 7%, *P* < 0.01), when compared to control (Figures 1(a) and 1(c)). Furthermore, the mRNA level of NF-200 was decreased (by 39 ± 12%, *P* < 0.05) already after 3 days exposure (4 DIV) to 20 *μ*M domoic acid (Figure 1(c)).

**Mature Cultures**

Exposure to the highest concentration of DomA (20 *μ*M) for 3 days induced a significant downregulation of the mRNA level of NF-68 (by 49 ± 15%, *P* < 0.05) as compared to control cultures (Figure 1(b)). The same concentration (20 *μ*M) decreased the NF-68 mRNA level further (by 72 ± 7%, *P* < 0.001) after the prolonged exposure for 10 days (Figure 1(b)).

In the case of NF-200, mRNA levels were also significantly decreased (by 65 ± 10%, *P* < 0.01) after exposure to 20 *μ*M domoic acid when compared to control cultures already after 3 days of exposure (Figure 1(d)). However, after prolonged (10 days) exposure, the decrease was already observed at the lower concentration (10 *μ*M) by 60 ± 9% (*P* < 0.05). (Figure 1(d)). The obtained results show that the neuronal cytoskeleton proteins are affected by DomA exposure in both immature and mature cultures of CGCs. However, the mature cultures seem to be more sensitive, since the effects were observed at an earlier time point compared to the immature ones.

### 3.2. The mRNA Expression of the NMDA- and GABA_A_-R Was Decreased by Domoic Acid While the mRNA Level of the AMPA-Receptor Was Unchanged

To determine whether the process of neuronal maturation was affected by DomA exposure, subunits of ionotropic receptors of the main excitatory neurotransmitter glutamate (AMPA- and NMDA-R) and a subunit of the GABA_A_ receptor, the main inhibitory neurotransmitter, were investigated. Subunits of the receptors were selected based on previous studies [17] to cover both early (NMDA-R) and later (GABA_A_-R and AMPA-R) expressed genes. 

**Figure 2:**
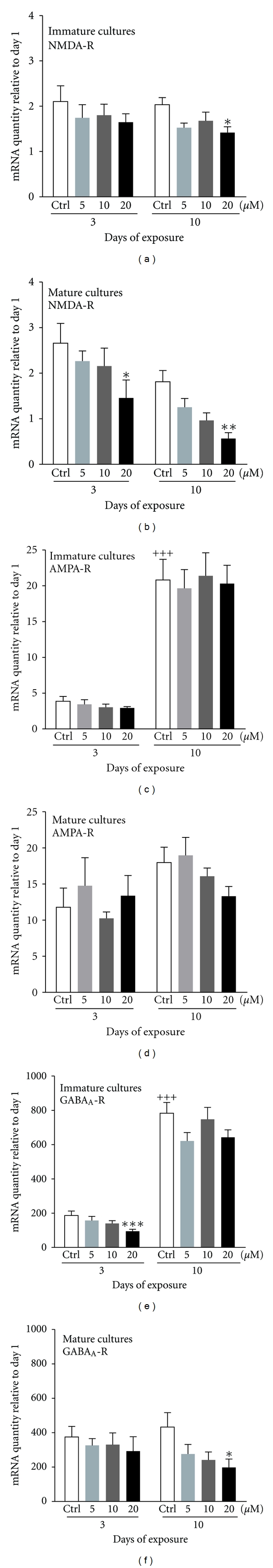
Changes in the mRNA levels of neural receptors in primary cultures of CGCs exposed to domoic acid (5 *μ*M, 10 *μ*M, and 20 *μ*M), at 1 DIV (immature cultures) or at 7 DIV (mature cultures) for 3 or 10 days. Note the significant downregulation of (a) NMDA-R in immature cultures after 10 days of exposure (20 *μ*M), (b) NMDA-R in mature cultures, (e) GABA_A_-R in immature cultures after 3 days of exposure (20 *μ*M), and (f) GABA_A_-R in mature cultures after 10 days of exposure (20 *μ*M). There were no changes for (c) AMPA-R in immature cultures and (d) AMPA-R in mature cultures. Gene expression levels were normalized to the standard calibrator, the housekeeping gene 18S rRNA and the mRNA expression at 1 DIV. Data represent mean ± S.E.M. of three independent experiments performed in duplicates. ^+^ *P* < 0.05  ^++^ *P* < 0.01  ^+++^ *P* < 0.001 comparing control cultures at 3 and 10 days. *P < 0.05  **P < 0.01  ***P < 0.001 comparing treated to control (untreated) cultures.

Based on the published studies, it is not clear whether DomA toxicity is mediated only by the AMPA-receptor or if the NMDA receptor could also play a role. In control (nontreated) cultures, a significant increase of the mRNA levels of both the AMPA-R (Figure 2(c)) and GABA_A_-R (Figure 2(e)) was observed between 3 and 10 days in the immature cultures, while the mRNA level remained stable in the mature cultures (Figures 2(d) and 2(f)). The mRNA level of the NMDA-R did not change during this time interval in either immature or mature control cultures (Figures 2(a) and 2(b)).

#### 3.2.1. Effects of Domoic Acid

**Immature Cultures**

After 10 days of exposure to the highest concentration of DomA (20 *μ*M), the mRNA level of the NMDA-R significantly decreased (by 30 ± 6%, *P* < 0.05) as compared to control cultures (Figure 2(a)). The same DomA concentration induced a significant downregulation of the mRNA level of the GABA_A_-R (by 49 ± 6%, *P* < 0.001) after 3 days of exposure (Figure 2(e)). However, after prolonged treatment of 10 days, no significant effect was observed at the mRNA level of the GABA_A_-R. Interestingly, the mRNA level of the AMPA-R was not changed after the DomA treatment at any time points (3 and 10 days) (Figure 2(c)).

**Mature Cultures**

In our mixed neuronal-glial cell culture model, the mRNA level of the NMDA-R seemed to be more affected in the mature cultures compared with the immature ones. 3 days of exposure to 20 *μ*M DomA significantly decreased the mRNA level of this receptor (by 45 ± 15% (*P* < 0.05) (Figure 2(b)), while no effect was observed in the immature cultures (Figure 2(a)). 10 days exposure to 20 *μ*M DomA further decreased the mRNA level of the NMDA-R (by 69 ± 7%, *P* < 0.01) (Figure 2(b)). The mRNA level of the GABA_A_-R was reduced only after 10 days exposure to the highest concentration of 20 *μ*M DomA (54 ± 11%, *P* < 0.05) (Figure 2(f)). However, no effect was observed at the mRNA level of the AMPA-R (Figure 2(d)).

These results show that the mRNA levels of the NMDA- and GABA_A_-R, but not the mRNA level of the AMPA-R, were downregulated in both immature and mature cultures exposed to domoic acid, however, in different ways. Once again, the mature cultures seem to be more sensitive to DomA as the observed effects were stronger and took place earlier especially in the case of the mRNA of the NMDA-R (Figures 2(a) and 2(b)).

### 3.3. Domoic Acid Exposure Affected the mRNA Levels of the Astrocytic Markers (GFAP and S100*β*) in Mature but not in Immature Cultures

**Figure 3:**
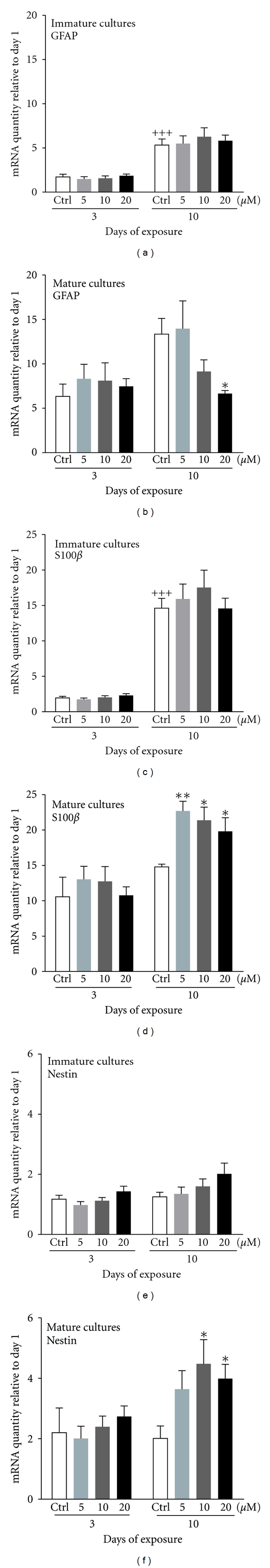
Changes in the mRNA levels of astrocytic and neural precursor markers in primary cultures of CGCs exposed to domoic acid (5 *μ*M, 10 *μ*M, and 20 *μ*M) at 1 DIV (immature cultures) or at 7 DIV (mature cultures) for 3 or 10 days. There were no observed changes for (a) GFAP in immature cultures, (c) S100*β* in immature cultures, and (e) nestin expression in immature cultures. However, there was a significant decrease of (b) GFAP mRNA levels in mature cultures after 10 days of exposure (20 *μ*M) and increase of (d) S100*β* mRNA expression in mature cultures after 10 days of exposure and (f) nestin mRNA expression in mature cultures after 10 days of exposure. Gene expression levels were normalized to the standard calibrator, the housekeeping gene 18S rRNA and the mRNA expression at 1 DIV. Data represent mean ± S.E.M. of three independent experiments performed in duplicates.   ^+^P < 0.05  ^++^P < 0.01  ^+++^P < 0.001 comparing control cultures at 3 and 10 days. *P < 0.05  **P < 0.01  ***P < 0.001 comparing treated to control (untreated) cultures.

During development of the central nervous system the astrocytes play an important role, as they release trophic factors, guide axons, influence functional plasticity of synapses, and protect neurons from oxidative stress [23–26]. For this purpose, we have studied two markers of mature astrocytes, the intermediate filament GFAP and the zinc-calcium-binding protein S100*β*. In control cultures, the mRNA levels of GFAP and S100*β* significantly increased with time (between 3 and 10 days) in immature cultures (Figures 3(a) and 3(c)). In mature cultures the mRNA level of GFAP continued to increase, however, not significantly (Figure 3(b)), while the S100*β* remained stable (Figure 3(d)). 

Moreover, to cover earlier neural developmental stages, nestin, a marker for neural precursor cells, was studied. Nestin is a cytoskeleton protein mainly expressed in neural precursor cells but it has also been reported to be re-expressed in activated astrocytes as a response to neuronal damage [27–29]. The mRNA expression of nestin was stable over the studied time points in both immature and mature control cultures (Figures 3(e) and 3(f)).

#### 3.3.1. Effects of Domoic Acid

**Immature Cultures**

Exposure to DomA up to 20 *μ*M did not induce any significant changes at the mRNA levels for the astrocytic markers GFAP and S100*β* (Figures 3(a) and 3(c)) or the neural precursor marker nestin (Figure 3(e)).

**Mature Cultures**

Interestingly, exposure of mature cultures to the highest concentration of DomA (20 *μ*M) for 10 days induced a significant downregulation of the mRNA expression of the astrocytic marker GFAP (by 50 ± 3%; P < 0.05) (Figure 3(b)), as compared to control cultures. In contrast, the mRNA level of S100*β* was upregulated after the prolonged exposure (10 days) at all studied concentrations (5, 10, and 20 *μ*M) by 53 ± 9% (P < 0.01), 45 ± 13% (P < 0.05) and 34 ± 13% (P < 0.05), respectively, (Figure 3(d)).

Furthermore, the mRNA levels of nestin in mature cultures significantly increased after 10 days exposure to 10 and 20 *μ*M DomA (up to 123 ± 40%, P < 0.05 and 98 ± 24%, P < 0.05, resp.) (Figure 3(f)). The increase in the mRNA expression of nestin could be due to proliferation of precursor cells, higher expression of nestin per cell, or, and most likely, because of re-expression of nestin in astrocytes that probably became activated in response to DomA-induced neurotoxicity.

The obtained results indicate that mature astrocytes were affected by the exposure to domoic acid, as the gene expression of the various astrocytic markers was down- (GFAP) or upregulated (S100*β* and nestin). There were no observed changes in the immature cultures.

### 3.4. The AMPA/KA Receptor Mediates DomA-Induced Neurotoxicity in both Immature and Mature Cultures of CGCs

**Figure 4:**
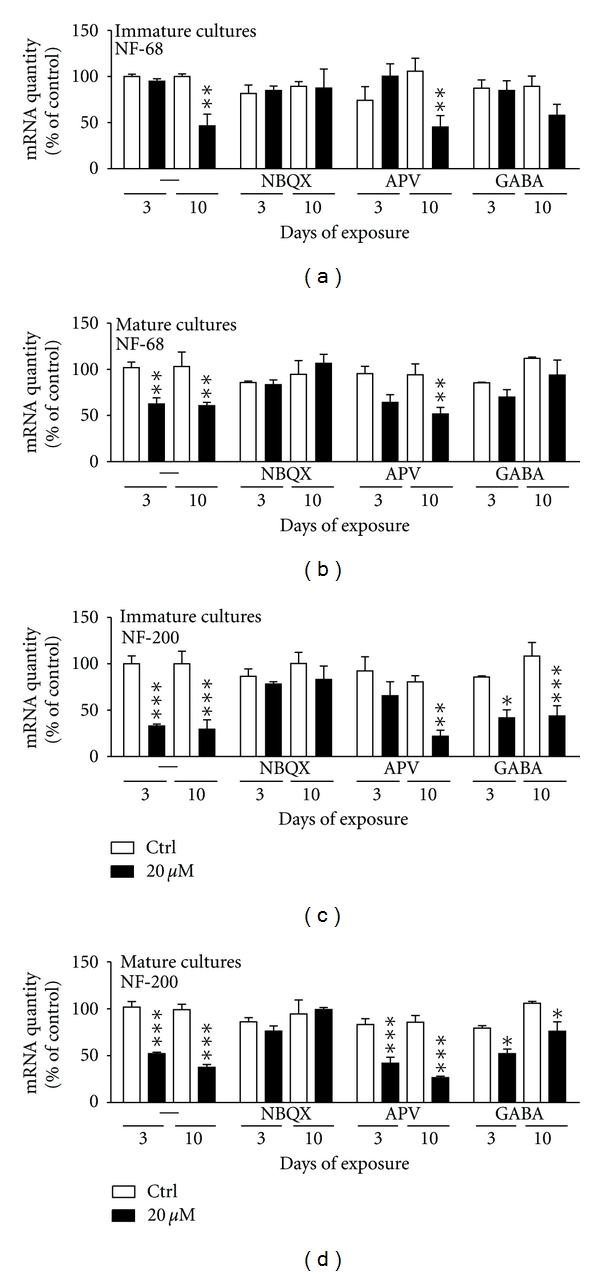
Changes in the mRNA levels of NF-68 and 200 in primary cultures of CGCs exposed to 20 *μ*M domoic acid alone or in combination with antagonists for the AMPA-R (NBQX, 20 *μ*M), NMDA-R (APV, 100 *μ*M) or the neurotransmitter GABA (10 *μ*M) at 1 DIV (immature cultures) or at 7 DIV (mature cultures) for 3 or 10 days. Note that NBQX but not APV prevent the domoic acid-induced downregulation of (a) NF-68 in immature cultures, (b) NF-68 in mature cultures, (c) NF-200 mRNA expression in immature cultures, and of (d) NF-200 in mature cultures. Gene expression levels were normalized to the standard calibrator, the housekeeping gene 18S rRNA and the mRNA expression at 1 DIV. Data represent mean ± S.E.M. of three independent experiments performed in duplicates.   *P < 0.05  **P < 0.01  ***P < 0.001 comparing treated to control (untreated) cultures.

**Figure 5:**
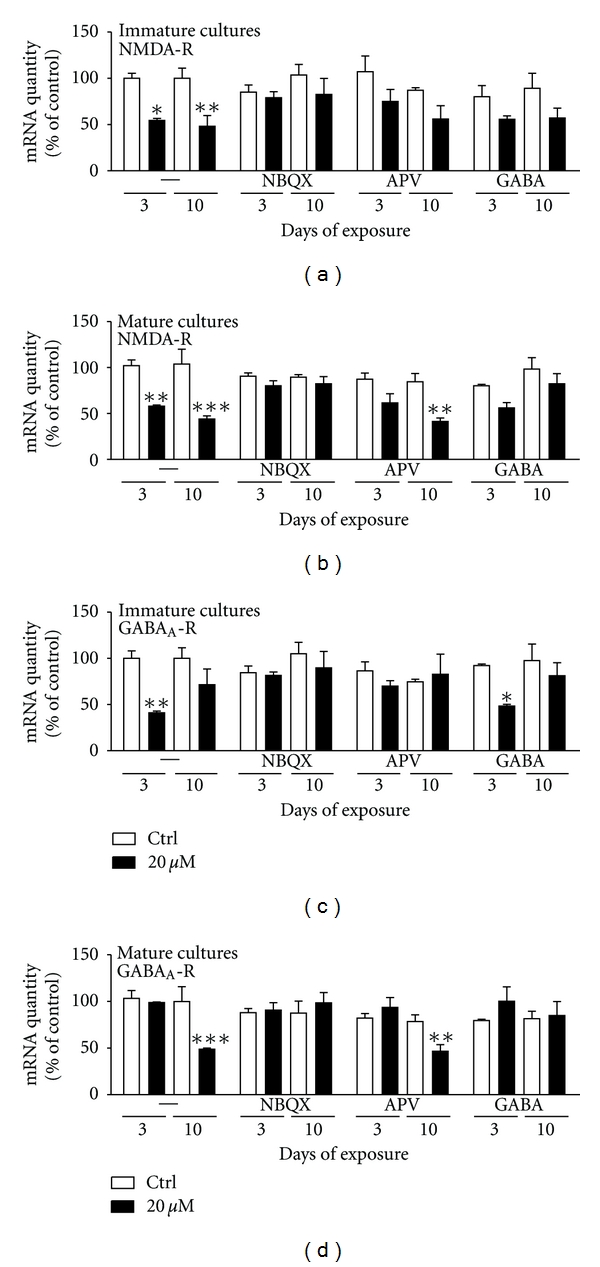
Changes in the mRNA levels of neural receptors in primary cultures of CGCs exposed to 20 *μ*M domoic acid alone or in combination with antagonists for the AMPA-R (NBQX, 20 *μ*M), NMDA-R (APV, 100 *μ*M), or the neurotransmitter GABA (10 *μ*M) at 1 DIV (immature cultures) or at 7 DIV (mature cultures) for 3 or 10 days. Note that NBQX completely and APV and GABA partly prevent the domoic acid induced downregulation of (a) NMDA-R in immature cultures, (b) NMDA-R in mature cultures, (c) GABA_A_-R mRNA expression in immature cultures, and of (d) GABA_A_-R in mature cultures. Gene expression levels were normalized to the standard calibrator, the housekeeping gene 18S rRNA and the mRNA expression at 1 DIV. Data represent mean ± S.E.M. of three independent experiments performed in duplicates.   *P < 0.05  **P < 0.01  ***P < 0.001 comparing treated to control (untreated) cultures.

**Figure 6:**
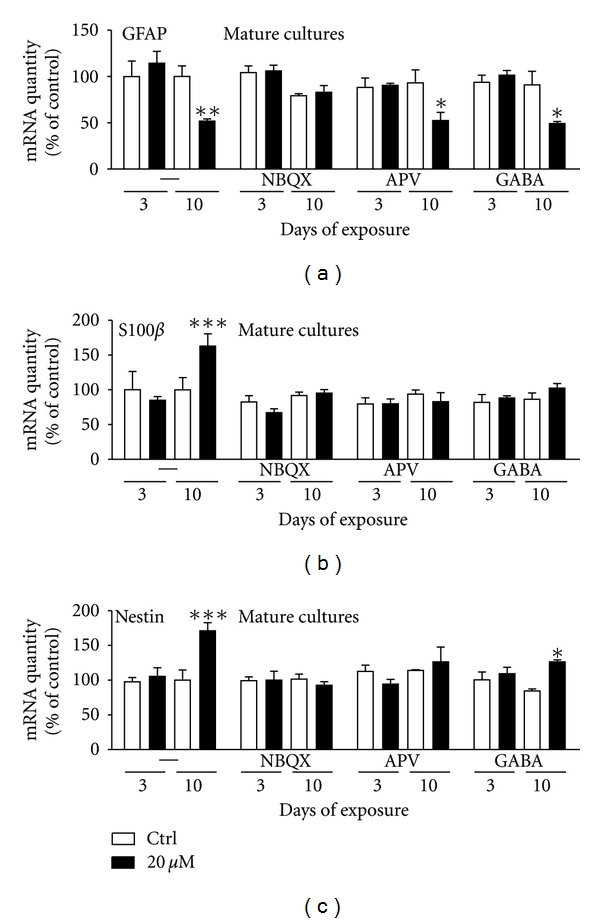
Changes in the mRNA levels of astocytic and precursor markers in mature primary cultures of CGCs exposed to 20 *μ*M domoic acid alone or in combination with antagonists for the AMPA-R (NBQX, 20 *μ*M), NMDA-R (APV, 100 *μ*M) or the neurotransmitter GABA (10 *μ*M) at 7 DIV for 3 or 10 days. NBQX but not APV or GABA prevented the domoic acid induced downregulation of (a) GFAP mRNA expression. Note that NBQX, APV and GABA could prevent the domoic acid induced up-regulation of (b) S100*β* and (c) nestin mRNA levels. Gene expression levels were normalized to the standard calibrator, the housekeeping gene 18S rRNA and the mRNA expression at 1 DIV. Data represent mean ± S.E.M. of three independent experiments performed in duplicates.   *P < 0.05  **P < 0.01  ***P < 0.001 comparing treated to control (un-treated) cultures.

In order to evaluate if the observed toxic effects induced by domoic acid were mediated by activation of the AMPA/KA- and/or the NMDA receptor, the cultures were treated with competitive antagonists (NBQX and APV, resp.) alone or in combination with the highest tested concentration of DomA (20 *μ*M). Additionally, to determine whether increased levels of the inhibitory neurotransmitter GABA could prevent domoic acid-induced excitotoxicity, as it has been proposed in *in vivo* studies [8], the cultures were pretreated with this neurotransmitter. The studied concentrations of the NMDA and AMPA/KA receptor antagonists (100 *μ*M of APV and 20 *μ*M of NBQX) and the agonist (10 *μ*M of GABA) were selected based on findings from the literature [9, 14, 30]. These concentrations of APV, NBQX, and GABA did not induce any changes in the mRNA levels of studied genes in the control cultures (Figures 4–6).

### 3.5. Antagonists of the AMPA/KA- but Not of the NMDA-R Prevented the Domoic Acid Induced Decrease of NF-68 and NF-200 mRNA Levels

#### 3.5.1. Immature Cultures

Exposure of the immature cultures to 20 *μ*M domoic acid for 10 days significantly decreased the mRNA levels of NF-68 (by 54 ± 13%; P < 0.01) (Figure 4(a)) compared to control cultures. Pretreatment with the AMPA/KA-R antagonist (20 *μ*M NBQX) completely prevented this effect, as the expression of the mRNA of NF-68 was at the same level as in the control cultures (Figure 4(a)). In contrast, pretreatment with the NMDA-R antagonist (100 *μ*M APV) did not have any effect, and the addition of GABA (10 *μ*M) partly prevented domoic acid-induced changes at the mRNA levels of NF-68 (Figure 4(a)). 

Similar results were observed in the case of the NF-200 mRNA, levels. Domoic acid alone downregulated the gene expression by 67 ± 2% (P < 0.001) after 3 days of exposure and by 71 ± 10% (P < 0.001) after 10 days compared to the control cultures (Figure 4(c)). The presence of the AMPA/KA-R (NBQX) antagonist completely prevented the DomA-induced changes of the NF-200 mRNA since it was brought up to the control level. Interestingly, the antagonist for the NMDA-R (APV) blocked the DomA-induced decrease of the NF-200 mRNA after 3 days of exposure but not after prolonged treatment (up to 10 days) (Figure 4(c)). Treatment with GABA did not have any effect on the domoic acid induced downregulation of the NF-200 mRNA levels (Figure 4(c)). 

#### 3.5.2. Mature Cultures

In mature cultures, the exposure to 20 *μ*M domoic acid alone downregulated the mRNA levels of NF-68 by 39 ± 7% (P < 0.01) after 3 days of exposure and by 41 ± 3% (P < 0.01) after 10 days (Figure 4(b)). When the AMPA/KA-R antagonist (NBQX) was present, the observed changes were prevented (Figure 4(b)). The NMDA-R antagonist (APV) did not have any effect as the mRNA expression of NF-68 was still decreased (by 33 ± 9% after 3 days of exposure and by 45 ± 7%, P < 0.01 after 10 days) as compared to control cultures (Figure 4(b)). Interestingly, the increased levels of the neurotransmitter GABA prevented the domoic acid effect, as the mRNA expression of NF-68 was at the same level as in the control cultures (Figure 4(b)). The mRNA level of NF-200 was downregulated by the 20 *μ*M DomA treatment in mature cultures after 3 days of exposure (by 49 ± 1%, P < 0.001) and after 10 days (by 62 ± 3%, P < 0.001) (Figure 4(d)). Pretreatment with NBQX prevented this effect, while the changes remained in the presence of APV (the mRNA expression of NF-200 was still decreased) (Figure 4(d)). GABA reduced the effects of DomA, as the mRNA levels of NF-200 decreased less (by 34 ± 6%, P < 0.05) after 3 and 10 days of exposure (by 28 ± 10%, P < 0.05) compared to treatment with domoic acid alone (by 49 ± 1%, P < 0.001 after 3 days and by 62 ± 3%, P < 0.001 after 10 days) (Figure 4(d)). 

The obtained results indicate that DomA-induced effects were mediated mainly by the AMPA/KA-R, as its antagonist NBQX could prevent the decrease observed at the mRNA levels for NF-68 and NF-200 in both immature and mature cultures. Furthermore, the inhibitory neurotransmitter GABA could also be involved, since increased levels partially protected the DomA-induced neurotoxicity, especially in the case of mature cultures. 

### 3.6. The AMPA/KA-R Antagonist Prevented Domoic Acid-Induced Decrease of NMDA- and GABA_A_-R mRNA Levels

#### 3.6.1. Immature Cultures

Domoic acid exposure (20 *μ*M) alone in immature cultures decreased the mRNA levels of the NMDA-R by 46 ± 2% (P < 0.05) after 3 days of exposure and by 52 ± 12% (P < 0.01) after 10 days compared to control cultures (Figure 5(a)). NBQX (antagonist for the AMPA/KA-R) completely prevented the observed decrease of the NMDA mRNA expression, while the addition of GABA and APV (NMDA-R antagonist) reduced the effect. 

Domoic acid exposure downregulated the GABA_A_-R mRNA expression by 59 ± 2% (P < 0.01) after 3 days of exposure versus control (Figure 5(c)). Interestingly, this effect was completely prevented by NBQX and APV (Figure 5(c)) indicating that both receptors, AMPA/KA-R and NMDA-R, were involved. However, increased levels of GABA itself did not have any effect, as the GABA_A_-R mRNA levels were still downregulated (by 47 ± 2%, P < 0.05) after 3 days of exposure (Figure 5(c)).

#### 3.6.2. Mature Cultures

Exposure of mature cultures to 20 *μ*M DomA-induced a significant downregulation of the NMDA-R mRNA levels at both time points 3 days of exposure (by 43 ± 1%, P < 0.01) and 10 days of exposure (by 58 ± 3%, P < 0.001) (Figure 5(b)). These effects were blocked by the AMPA/KA-R antagonist (NBQX), and the neurotransmitter GABA (Figure 5(b)), while the NMDA-R antagonist (APV) did not have any effect.

The mRNA expression of the GABA_A_-R was also significantly decreased by 20 *μ*M DomA (51 ± 1%, P < 0.001) after 10 days of exposure (Figure 5(d)). This decrease was prevented by NBQX and by GABA treatment (Figure 5(d)). However, the treatment with APV did not have any effect, as the expression of the GABA_A_-R was still significantly decreased (Figure 5(d)).

These results show that the AMPA/KA-R played a crucial role in DomA-induced toxicity, as the antagonist (NBQX) completely prevented the observed decrease in mRNA expression of the NMDA- and GABA_A_-R in both immature and mature cultures. Moreover, the NMDA-R was less involved, as its antagonist (APV) did not fully protect against the observed effects (Figure 5). Interestingly, the neurotransmitter GABA could protect against the domoic acid-induced toxicity in the mature cultures (Figures 5(b) and 5(d)) but not in the immature ones (Figures 5(a) and 5(c)). This could be due to the fact that the immature cultures could express lower levels of the functional GABA receptors at early time points in comparison to mature cultures.

### 3.7. Domoic Acid Induced Alternations at the mRNA Level of GFAP, S100*β*, and Nestin Were Partly Prevented by NBQX, APV or GABA

#### 3.7.1. Immature Cultures

Since in the case of immature cultures DomA did not induce any effects at the mRNA levels of the astrocytic markers, GFAP, and S100*β* or the neural precursor cell marker nestin, no further studies were performed with the antagonists of NMDA-R, AMPA/KA-R or with the neurotransmitter GABA.

#### 3.7.2. Mature Cultures

The mature cultures exposed to domoic acid (20 *μ*M) alone showed a significant downregulation of the mRNA levels of GFAP (by 48 ± 2%, P < 0.01) compared to control after 10 days of exposure (Figure 6(a)). These toxic effects were blocked by NBQX but neither by APV nor the neurotransmitter GABA, as the GFAP expression was still decreased after 10 days of exposure (by 44 ± 9%; P < 0.05 after APV treatment and by 46 ± 2%; P < 0.05 in the presence of GABA) (Figure 6(a)). In contrast to GFAP, 20 *μ*M DomA exposures induced significant upregulation by 63 ± 18% (P < 0.001) of the S100*β* mRNA levels after 10 days of exposure (Figure 6(b)). Interestingly, this effect was completely blocked by the pretreatment of NBQX, APV, or GABA (Figure 6(b)). 

Similarly to S100*β*, the mRNA expression of nestin (neural precursor cell marker) was significantly increased (by 71 ± 12% (*P* < 0.001) after 10 days of exposure to 20 *μ*M domoic acid when compared to control cultures (Figure 6(c)). This increase is likely due to re-expression of nestin in activated astrocytes. Both NBQX and APV blocked this effect as no differences in comparison to control cultures were observed (Figure 6(c)). The increased level of neurotransmitter GABA reduced the toxicity, as the effect was less significant (26%  ± 3%; *P* < 0.05) when compared to cultures exposed only to DomA (71%  ± 12%, *P* < 0.001) (Figure 6(c)).

Our results suggest that the various toxic effects induced by domoic acid in neuronal and glial cells of mixed primary culture of CGCs seem to be mediated by different receptors. The changes in mRNA expression of the neuronal markers (NF-68 and −200, and NMDA and GABA receptors) could mainly be prevented by NBQX, suggesting that the AMPA/KA receptor was involved. The effects observed at the level of astrocytic markers S100*β* and nestin (possibly as a marker of reactive astrocytes) could be prevented by both NBQX and APV, indicating that both AMPA/KA- and NMDA- receptors could play a role in DomA-induced toxicity. However, more studies should be performed to determine whether the astrocytic toxicity could be a secondary effect due to the decreased neurotoxicity observed in the presence of NBQX and APV.

## 4. Discussion

In this study, we have shown that domoic acid exposure-induced changes in the mRNA level of selected genes identified as specific markers for glial and neuronal cells in both developing (immature) and in mature mixed neuronal-glia cultures of primary rat CGCs. Interestingly, the changes in mRNA levels indicate that the mature cultures seemed to be more sensitive to the DomA exposure than the immature ones. In mature cultures, all neuronal markers (NF-68, NF-200, NMDA-R, and GABA_A_-R) were significantly and earlier downregulated after domoic acid exposure than in immature cultures, which indicates a higher vulnerability. 

These results suggest that the long-term exposure could lead to neuronal dysfunction, as the cytoskeleton proteins (both NF-68, and NF-200) as well as the expression of critical receptors both excitatory (NMDA) and inhibitory (GABA) were affected. The highest concentration of DomA also affected glia (decreased expression of GFAP and increased expression of S100*β*) that could additionally make neurons more vulnerable to DomA-induced toxicity. Also, higher expression of nestin supports the possible presence of activated astrocytes in response to DomA exposure.

The obtained results indicate that the applied gene expression tool is sensitive enough to identify cellular changes at concentrations that are not identified by cytotoxicity assays, such as AB used in these studies.

In the previous study by Qiu et al. [9], the obtained results support our findings. Indeed, it has been shown that NMDA receptor could play an important role as an increase in the excitotoxicity was observed with increasing *in vitro* time. This could be due to lower expression of the glutamate receptors (AMPA/KA and NMDA) in the younger cultures, which have been proposed to mediate the excitotoxicity of domoic acid. It has also been proposed that more Ca^2+^ permeable AMPA/KA-Rs or AMPA-R isoforms could be activated in the mature cultures in comparison to the younger cultures [9], causing glutamate release that subsequently could activate the NMDA receptor [10, 11] promoting neurotoxicity.

In our studies, the changes in the expression of the receptors during development could explain the increased vulnerability as the mRNA levels of the AMPA-R were increased approximately fivefold at 3 days and 20-fold at 10 days as compared to the levels at 1 day. In contrast to our observations,* in vivo* animal studies have clearly reported that fetuses and neonates are much more vulnerable to domoic acid-induced toxicity than adults [1, 3, 4]. The *in vivo* vulnerability of the immature brain might depend more on the increased bioavailability of the compound due to incomplete blood brain barrier formation and decreased serum clearance than the DomA-induced mechanism of toxicity itself. This could explain the contrary *in vitro* finding, where mature cultures seem to be more susceptible than immature ones to DomA-induced neurotoxicity.

The expression of these two receptors (AMPA and NMDA) should be evaluated in whole animal studies to make a better comparison between *in vitro* and *in vivo* DomA-induced effects to be able to identify the critical stages of brain development that are more vulnerable to domoic acid exposure. Such knowledge would then help to identify whether a specific age group might be of greater risk to domoic acid-induced neurotoxic effects than others. If DomA toxicity is only mediated through the glutamate receptors, the lack of these functional receptors during the first DIV would be responsible for lower levels of toxicity induced by DomA as observed in our studies. 

Domoic acid-induced neurotoxicity, in both mature and immature cultures, seemed to be mediated mainly through the AMPA/KA-R as NBQX completely reversed the decreased mRNA levels for all the selected neuronal markers (NF-68, NF-200, NMDA-R, and GABA_A_-R). In contrast, the NMDA-R antagonist (APV) could not prevent all the changes of these mRNA levels. 

In acute *in vitro* studies (1 hr of exposure), it have been shown that AMPA/KA receptors (not NMDA-R) mediate low concentration domoic acid- (0.1 *μ*M) induced apoptosis, while a higher concentration (10 *μ*M) activates both the AMPA/KA-R and NMDA receptor leading to necrosis [31]. 

Interestingly, in this studies, the toxicity of DomA (changes in the expression of NF-68, NF-200, NMDA-R, GABA_A_-R, S100*β*, and nestin) was reduced in mature culture by adding external GABA. *In vivo* studies have shown reduced expression of the neurotransmitter GABA in the brain after domoic acid exposure [3, 8, 32] and a similar decrease could possibly also take place in our *in vitro* model. Indeed, local hippocampal administration of GABA in rat *in vivo* studies resulted in neuronal protection against DomA- induced toxicity [8] as well as in this *in vitro* study. Moreover, in our previous *in vitro* study, we observed that the function of the GABA_A_ receptor was downregulated after long-term exposure to DomA, possibly due to lower levels of the neurotransmitter GABA [33]. In contrast, GABA did not reduce the toxic effects of domoic acid in immature cultures perhaps due to the low expression of GABA receptors. 

The obtained results also pointed out to the important role of glia in DomA-induced neurotoxicity. In the previous studies, including the results obtained by Giordano et al. [31], pure neuronal cultures were applied, while our experiments were performed using mixed neuronal-glia cultures (neurons, astrocytes, and microglia), and especially, the presence of astrocytes could play an important role in the DomA-induced mechanisms of toxicity. This is a critical issue, as neurons respond differently to the same toxicant depending on the presence or absence of glia [34]. There are some reports suggesting that the domoic acid toxicity could be enhanced in the presence of astrocytes and microglia due to for instance DomA-induced inhibition of glutamate uptake by astrocytes [15] followed by glial activation (initially of microglia and later of astrocytes). Activated microglia and astrocytes could release neurotoxic free oxygen radicals, nitric oxide and proinflammatory cytokines [16, 35, 36]. Indeed, in mixed neuronal-glia control primary cultures of CGCs, the proliferation of astrocytes and microglia takes place over time [18], and DomA-induced increased toxicity observed in mature cultures could, therefore, be due to a higher amount of glial cells (both astrocytes and microglia). The immature cultures (4 DIV) consist of 93 ± 3% neurons, 4 ± 0.3% astrocytes, and 3 ± 0.1% microglia, while the mature cultures (8–12 DIV) consist of 78 ± 3% neurons, 18 ± 0.8% astrocytes, and 4 ± 0.2% microglia [18].

In our studies, domoic acid clearly affected not only neurons, but also astrocytes as changes in the mRNA expression of GFAP (downregulation) and S100*β* (upregulation) was observed. However, these effects could only be observed in mature cultures, and the lack of effects in the immature cultures could be because of low levels of astrocytes. Moreover, the mature cultures showed a significant increase in the mRNA level of nestin after DomA exposure that has been reported to be a sensitive marker of activated astrocytes [37–39]. 

Decrease in GFAP expression has been associated with the inhibition of glutamate uptake [40, 41], which indeed could be the case in our study, as DomA exposure has shown to decrease uptake of glutamate in astrocytes [15]. In contrast, increased level of S100*β* is recognized as a marker of brain damage [42, 43]. While extracellular S100*β* at normal concentrations has a neurotrophic effect, higher concentrations can activate astrocytes and microglia and induce neuronal cell death [44]. Moreover, S100*β* has been linked to different brain pathological conditions such as Alzheimer's disease [45] and Down's syndrome [46, 47]. Similarly, the domoic acid-induced upregulation of nestin mRNA is likely due to glia activation in response to DomA induced neuronal damage (observed as decreased mRNA for the neuronal markers) rather than due to the proliferation of neural precursor cells. 

Summing up the obtained results suggests that DomA-induced neurotoxicity mediated through diverse mechanisms of toxicity in mature and immature cultures could be identified by the observed changes in mRNA expression of different cell markers (both neuronal and glial). 

Evaluation of gene expression could be a promising endpoint [17, 18] to be included in an *in vitro* DNT testing strategy. Such approach could be useful for an initial identification and further prioritization of compounds that might have DNT potential.
